# Identification of mitochondrial related gene characteristics and potential molecular mechanism of acute otitis media

**DOI:** 10.3389/fmolb.2025.1642269

**Published:** 2025-10-22

**Authors:** Dingjing Zi, Xiaoyong Ren

**Affiliations:** ^1^ Department of Otolaryngology, The Second Affiliated Hospital of Air Force Medical University, Xi’an, China; ^2^ Department of Otorhinolaryngology Head and Neck Surgery, The Second Affiliated Hospital of Xi’an Jiaotong University, Xi’an, China

**Keywords:** acute otitis media, mitochondria, immune infiltration, machine learning, inflammatory

## Abstract

**Background:**

Acute otitis media (AOM) is a prevalent pediatric infection worldwide, with mitochondrial dysfunction and immune responses implicated in its pathogenesis. However, the precise mechanisms remain elusive.

**Methods:**

Mitochondrial-related genes were extracted from Spn-AOM and NTHi-AOM datasets in the Gene Expression Omnibus (GEO). Differentially expressed mitochondrial genes (MitoDEGs) were identified and analyzed through functional enrichment analysis. Key MitoDEGs strongly linked to AOM were determined using least absolute shrinkage and selection operator (LASSO) and random forest (RF) models. Immune cell infiltration patterns were evaluated via the Cibersort algorithm, and associations between hub MitoDEGs and immune cells were examined. A regulatory network was established to elucidate gene regulation, and qRT-PCR validation was performed in C57BL/6 mice.

**Results:**

We identified 18 MitoDEGs in Spn-AOM and 14 in NTHi-AOM. Functional enrichment analysis highlighted their involvement in mitochondrial processes, peroxisomal activity, cell cycle control, and amino acid metabolism. LASSO and RF analyses pinpointed FAM110B, LIG1, and PDK1 as key genes. Immune infiltration analysis demonstrated significant associations between these genes and immune cell composition. TF-miRNA-mRNA network predictions suggested potential regulatory mechanisms.

**Conclusion:**

This study reveals mitochondrial gene expression alterations in AOM, identifying FAM110B, LIG1, and PDK1 as critical genes associated with immune cell infiltration. These findings provide insights into the mitochondrial-immune interplay in AOM pathogenesis.

## Introduction

Acute otitis media (AOM) is an inflammatory condition of the middle ear that develops rapidly and is marked by fluid accumulation, eardrum bulging or perforation with discharge, as well as symptoms such as fever and ear pain. The primary bacterial pathogens responsible for AOM are *Streptococcus* pneumoniae (Spn) and nontypeable *Haemophilus* influenzae (NTHi) (van den Broek et al., 2019; Avnstorp et al., 2016; Heidemann et al., 2016). As one of the most prevalent infections in children globally, AOM is a major contributor to pediatric antibiotic prescriptions (Vojtek et al., 2017; Paul and Moreno, 2020). However, the growing issue of antibiotic resistance necessitates the exploration of alternative strategies for both the prevention and treatment of this condition. Identifying novel therapeutic targets for AOM is therefore critical in addressing this challenge.

In recent years, mitochondria have emerged as key players in various diseases due to their central role in cellular energy metabolism and apoptosis (Rath et al., 2021). While conventionally regarded as energy-producing organelles, mitochondria are now recognized for their involvement in immune responses and inflammatory pathways. Specifically, mitochondrial components can act as DAMPs (damage-associated molecular patterns), triggering innate immune responses under conditions of cellular stress or injury (Kerur et al., 2018; Mills et al., 2017). Furthermore, mitochondria have been implicated in antiviral defense mechanisms, highlighting their importance in immune regulation (Vazquez and Horner, 2015). Notably, in addition to bacterial infections, AOM can also arise from viral pathogens such as respiratory syncytial virus, bocavirus, and adenovirus (Pettigrew et al., 2011). This underscores the need to investigate the role of mitochondrial regulation in AOM pathogenesis.

Emerging evidence indicates mitochondrial dysfunction and mitochondrial DNA damage in middle ear tissues of otitis media patients (Tatar et al., 2016; Jeyakumar et al., 2009). Additionally, mitochondria appear to influence the disease process and secretion formation in otitis media (Lee et al., 2021). Although some studies have explored the interplay between mitochondria and otitis media, research on their specific role in AOM remains unknown. Given the pivotal role of mitochondria in inflammatory processes, a deeper understanding of their regulatory mechanisms in AOM is essential. Elucidating mitochondrial functions in this context may enhance our understanding of AOM development and progression. This study seeks to investigate mitochondria-related genes in AOM through bioinformatics approaches, aiming to uncover novel targets for intervention and improve clinical outcome.

## Materials and methods

### The dataset used in this study

The study utilizes datasets GSE23140 (Yu et al., 2012) and GSE27990 (Ritchie et al., 2015), both obtained from the Gene Expression Omnibus (GEO) database (https://www.ncbi.nlm.nih.gov/geo/). These transcriptomic datasets were generated from peripheral blood mononuclear cells (PBMCs) isolated from heparinized peripheral venous blood samples collected from pediatric subjects (aged 6–30 months) during both the acute otitis media (AOM) episode and a pre-infection healthy baseline. The GSE23140 dataset includes four normal samples and 4 Spn-AOM samples, while the GSE27990 dataset consists of four normal samples and 4 NTHi-AOM samples. Additionally, mitochondrial-related genes were retrieved from the MitoCarta 3.0 database (http://www.broadinstitute.org/mitocarta) and the Gene Set Enrichment Analysis (GSEA) database (https://www.gsea-msigdb.org/gsea/index.jsp). By identifying the intersection of these gene sets, a final list of 2,030 mitochondrial-related genes was compiled.

### Identification of differentially expressed genes

We analyzed the GSE23140 and GSE27990 datasets to identify mitochondrial differentially expressed genes (MitoDEGs) in Spn-AOM and NTHi-AOM samples compared to normal utilizing the Limma package (Yu et al., 2012) in R statistical software (version 4.2.2). MitoDEGs were selected based on the criteria of |LogFC| > 0.5 and p-value <0.05.

### GO and KEGG functional enrichment analysis

To further investigate the potential functions of MitoDEGs, we conducted Gene Ontology (GO) and Kyoto Encyclopedia of Genes and Genomes (KEGG) functional enrichment analyses using the ClusterProfiler package [4.4.4] in R (Ritchie et al., 2015). GO analysis provided functional annotations across three categories: molecular function (MF), biological processes (BP), and cellular components (CC). KEGG analysis was used to explore molecular functions and the associated signaling pathways.

### Screening of hub genes

To identify hub genes, we utilized the Least Absolute Shrinkage and Selection Operator (LASSO) method and the Random Forest (RF) algorithm. LASSO was applied to filter candidate genes with binomial bias, using the “glmnet” package (version 4.1.7) in R for data analysis. This process involved determining variable lambda values, likelihood estimates, or classification error rates, followed by data visualization. The RF method, a robust approach for dataset prediction, was implemented using the “randomForest” package in R to rank predictive variables based on their importance. Genes identified through the intersection of both machine learning methods were designated as hub genes.

### Immune infiltration Cibersort

To infer immune cell composition within the samples, we performed deconvolution analysis using the ‘CIBERSORT’ algorithm (version 1.03) in R. This method applies support vector regression (SVR) to estimate the relative proportions of distinct immune cell types within bulk gene expression data. The pre-defined leukocyte signature matrix (LM22), comprising gene expression markers for 22 human immune cell subtypes—including T and B cells, plasma cells, natural killer cells, macrophages, monocytes, dendritic cells, and granulocytes—was obtained from the CIBERSORTx portal (https://cibersortx.stanford.edu/).

The CIBERSORT algorithm was run using default parameters and 1,000 permutations to compute significance estimates for each sample. Results with a deconvolution p-value of less than 0.05 were considered statistically reliable and retained for downstream analysis. The resulting immune cell proportions were used to evaluate differences in the immune microenvironment between AOM and control conditions. Furthermore, Spearman correlation analysis was conducted to assess potential associations between the expression levels of mitochondrial hub genes and specific immune cell subsets (Chen et al., 2018; Newman et al., 2015).

### Animals

C57BL/6 male mice were procured from Beijing Charles River Co., Ltd. and maintained for 8 weeks. Before experimentation, they were acclimated to local standard conditions (temperature: 20 °C–25 °C, humidity: 40%–70%) for at least 1 week. For *in vivo* studies, the mice were randomly assigned to AOM group and control (untreated) group. Each mouse in AOM group received an intraperitoneal (i.p.) injection of 5 μL *Streptococcus* pneumoniae serotype 19F suspension (1 × 10^7^ CFU/mL), while each control mice were administered an equal volume of Phosphate-Buffered Saline (PBS). After 24 h, all mice were euthanized via cervical dislocation, and middle ear tissues along with peripheral blood were collected. All procedures were conducted in accordance with the ARRIVE guideline and approved by the Ethics Committee of The Second Affiliated Hospital of Air Force Medical University.

### Histological analysis

Middle ear mucosal tissues were collected and fixed in 4% paraformaldehyde at room temperature for 12 h. The samples were subsequently dehydrated, embedded in paraffin, sectioned (4 µm thick), rehydrated, and stained with hematoxylin and eosin (H&E) using a Beyotime staining kit. The stained slides were examined under a BZ-X810 microscope to assess bacterial-induced pathological characteristics in the middle ear. Mucosal thickness was quantified in five randomly selected mice per group using ImageJ software. Investigators conducting the analysis were blinded to group allocation.

### RT-qPCR

Total RNA was extracted from serum samples using the RNAliquid Blood RNA Kit (Aidlab, China) following the manufacturer’s instructions. First-strand cDNA was synthesized from 1 µg of mRNA using the HiScript III first Strand cDNA Synthesis Kit. Quantitative real-time PCR (RT-qPCR) was performed on a CFX96 Touch System (Bio-Rad, USA) using the Taq Pro Universal SYBR qPCR Master Mix (Vazyme, China). Relative mRNA expression levels were calculated using the 2^–∆∆Ct method normalized through GAPDH. Primers were synthesized by Sangon (China), with specific sequences for mice listed in Table 1.

**TABLE 1:** The sequence of the primers.

Gene	Forward	Reverse
GAPDH	5′-GCCTCCTCCAATTCAACCCTT-3′	5′-CCAAATCCGTTCACACCGAC-3′
FAM110B	5′-ACATAGGCAAGGTGCATGGTT-3′	5′-AGGAGGTGTCGTGAACTGAG-3′
LIG1	5′-AGCAAGGTGACGTCATTGGT-3′	5′-GCATGTTGGCAGCAGAAGTC-3′
PDK1	5′-ACTGCAGACAGCTTACCTGA-3′	5′-AGGCAACTCTTGTCGCAGAA-3′

### Enzyme-linked immunosorbent assay (ELISA)

Serum levels of IL-1β, TNF-α, and IL-6 were quantified using ELISA kits (Beyotime, China) in accordance with the manufacturer’s instructions. Briefly, 50 µL of serum was added to each well of a 96-well plate and incubated at 37 °C for 30 min, with a blank control included. Subsequently, 100 µL of freshly diluted enzyme-labeled antibody was applied and incubated under the same conditions. Following this, 50 µL of TMB substrate solution was added and allowed to react at 37 °C for 10–30 min. Then, 50 µL of sulfuric acid was added per well. Optical density (OD) was recorded at 450 nm using a Multiskan™ FC Microplate Photometer.

### Statistical analysis

Bioinformatic analyses were conducted using R software (version 4.2.2). The Wilcoxon rank-sum test was applied to evaluate differences in quantitative variables, while Spearman correlation analysis was utilized to explore associations between diagnostic gene expression and infiltrating immune cells. For statistical evaluation of experimental data, GraphPad Prism (version 9.4.0) was used. Differences between the control and AOM groups were assessed using an independent Student’s t-test. All statistical analyses were two-tailed, with a significance threshold set at p < 0.05.

## Results

### Machine learning-based screening of MitoDEGs

**FIGURE 1:**
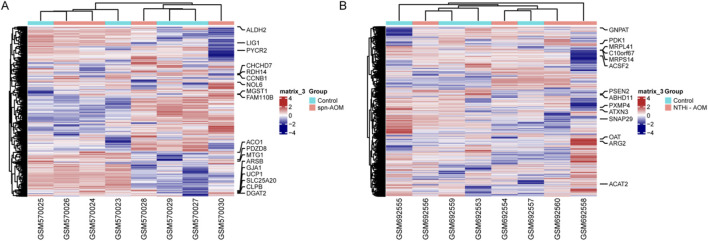
MitoDEGs Across normal and AOM. **(A)** Heatmap displays the MitoDEGs between normal samples (labeled with blue, annotated as “Normal”) and Spn-AOM samples (labeled with red, annotated as “AOM”). **(B)** Heatmap displays the MitoDEGs between normal samples (labeled with blue, annotated as “Normal”) and NTHi-AOM samples (labeled with red, annotated as “AOM”).

The GSE23140 dataset, containing gene expression data from four healthy and 4 Spn-AOM samples, and the GSE27990 dataset, comprising gene expression data from four healthy and 4 NTHi-AOM samples, were analyzed to screen for hub MitoDEGs via machine learning. In GSE23140, a total of 1,376 mitochondria-related genes were selected after preprocessing, then 18 MitoDEGs were identified, which includes 11 activated genes and seven suppressed genes (Figure 1A). In GSE27990, a total of 1713 mitochondria-related genes were selected, among which 14 showed statistical significance (Figure 1B). These included five upregulated genes (OAT, ARG2, ACTA2, PDK1 and GNPA7) and nine downregulated genes (MRPL41, C10orf67, MRPS14, ACSF2, PSEN2, ABHD11, PXMP4, ATXN3, SNAP29). These findings indicate a potential role of MitoDEGs in the pathogenesis of AOM.

### Underlying molecular function of MitoDEGs in different subtypes of AOM

**FIGURE 2:**
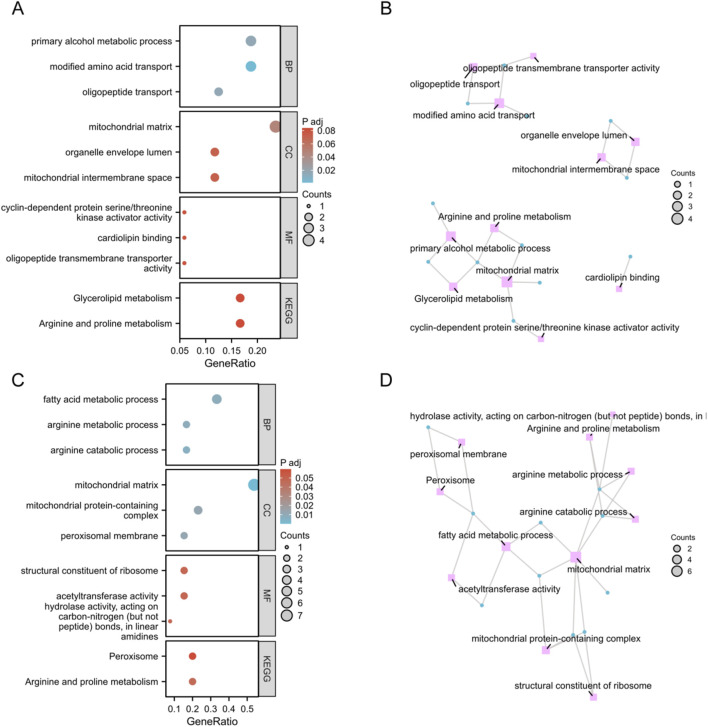
Functional Enrichment Analysis of Spn-AOM and NTHi-AOM. **(A)** Bubble plots of GO and KEGG (Spn-AOM). **(B)** Network diagrams of GO and KEGG (Spn-AOM). **(C)** Bubble plots of GO and KEGG (NTHi-AOM). **(D)** Network diagrams of GO and KEGG (NTHi-AOM).

To investigate the potential role of MitoDEGs in AOM, we conducted functional annotation using GO and KEGG pathways. As illustrated in Figure 2A, in Spn-AOM, biological process analysis revealed significant enrichment in modified amino acid transport, oligopeptide transport, and primary alcohol metabolism. In terms of cellular component, MitoDEGs were notably associated with the mitochondrial matrix, mitochondrial intermembrane space, and organelle envelope lumen. Regarding molecular function, the enriched terms mainly contained oligopeptide transmembrane transporter activity, cardiolipin binding, and cyclin-dependent protein kinase activator activity. Likewise, KEGG result suggested a strong connection to arginine and proline metabolism, as well as glycerolipid metabolism. Additionally, the network visualization revealed multiple key vectors and interactions, underscoring the intricate relationships between these biological events in Spn-AOM (Figure 2B).

For NTHi-AOM, BP analysis highlights significant involvement in arginine catabolic process, arginine metabolism, and fatty acid metabolism. In CC, MitoDEGs were primarily linked to the mitochondrial matrix, mitochondrial protein-containing complexes, and the peroxisomal membrane. MF analysis identified associations with acetyltransferase activity, ribosomal structural constituents, and hydrolase activity acting on carbon-nitrogen bonds in linear amidines. KEGG pathway enrichment reinforces these findings, emphasizing arginine metabolism and peroxisome activity, thereby supporting the biological significance of these pathways in NTHi-AOM (Figures 2C,D). Collectively, the enrichment analyses point to mitochondria-related genes playing a central role in regulating AOM.

### Identifications of hub MitoDEGs via machine learning

**FIGURE 3:**
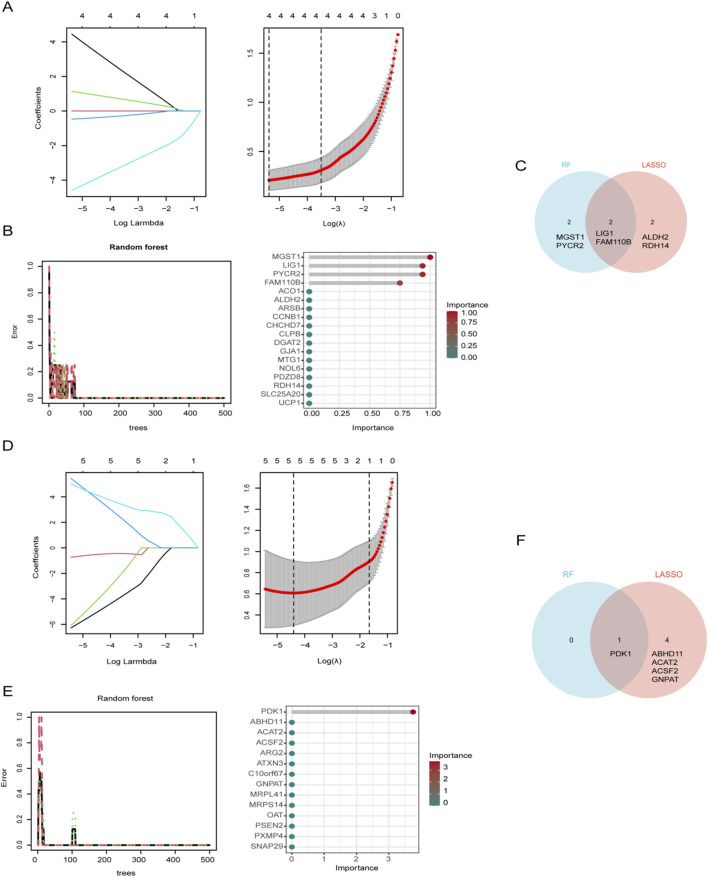
Identification of Mitochondrial biomarkers. **(A)** LASSO regression coefficient path diagram and cross-validation curve for Spn-AOM. **(B)** Impact of decision tree count on error rate and variable importance assessment for 18 MitoDEGs in Spn-AOM. **(C)** Venn diagram illustrating gene overlap between two algorithms for Spn-AOM. **(D)** LASSO regression coefficient path diagram and cross-validation curve for NTHi-AOM. **(E)** Impact of decision tree count on error rate and variable importance assessment for 14 MitoDEGs in NTHi-AOM. **(F)** Venn diagram illustrating gene overlap between two algorithms for NTHi-AOM.

LASSO regression and RF analysis were applied to the previously identified differentially expressed genes to further refine key candidates. In Spn-AOM, LASSO regression reduced the 18 MitoDEGs to a subset of four genes (LIG1, FAM110B, ALDH2, and RDH14) (Figure 3A), while RF analysis identified another set of four key genes (LIG1, FAM110B, MGST1, and PYCR2) (Figure 3B). A Venn diagram was used to determine the overlap between the two methods, revealing two common genes, LIG1 and FAM110B (Figure 3C).

Likewise, LASSO regression narrowed the 14 MitoDEGs down to five (PDK1, ABHD11, ACAT2, ACSF2, and GNPAT) in NTHi-AOM (Figure 3D), whereas RF analysis pinpointed a single key gene, PDK1 (Figure 3E). The intersection of LASSO and RF results, visualized using a Venn diagram, identified PDK1 as the sole overlapping gene (Figure 3F). These findings highlight potential biomarkers for different subtypes of AOM, offering valuable insights for further research into its molecular mechanisms.

### Expression patterns of biomarkers in AOM

**FIGURE 4:**
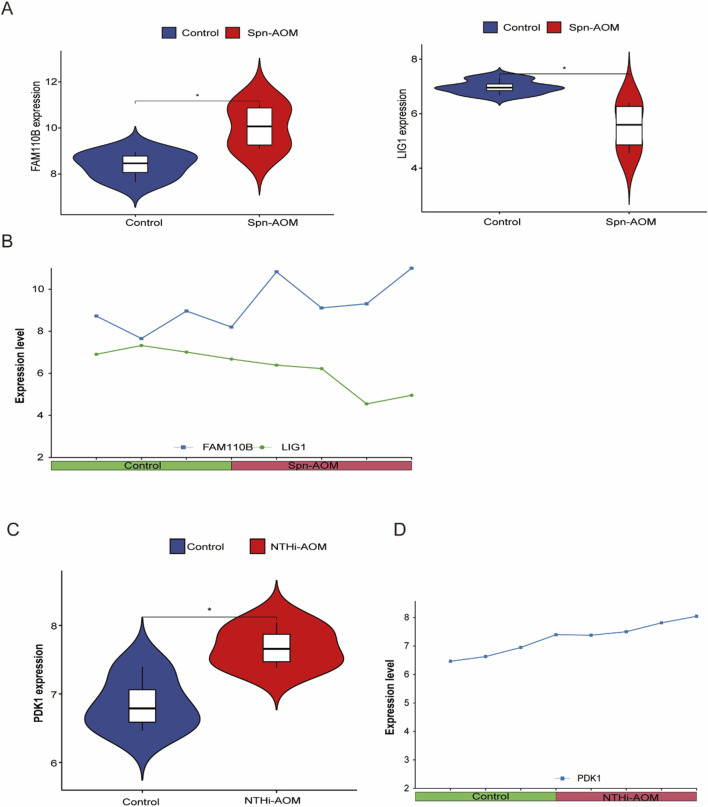
Hub Gene Expression in AOM. **(A)** Violin plot showing the differential expression of hub genes in Spn-AOM; **(B)** Line graph depicting the expression of hub genes in Spn-AOM; **(C)** Violin plot illustrating the differential expression of hub genes in NTHi-AOM; **(D)** Line graph displaying the expression of hub genes in NTHi-AOM.

We validated the expression patterns of identified key genes through direct comparative analysis between the control (pre-infection, healthy) and AOM groups within their respective datasets. For Spn-AOM, the expression levels of FAM110B and LIG1 were compared between the four control samples and 4 Spn-AOM samples in the GSE23140 dataset, revealing that FAM110B showed increased expression, while LIG1 exhibited downregulation (Figure 4A). These findings were corroborated in Figure 4B, illustrating the activation of FAM110B and suppression of LIG1 in Spn-AOM. Similarly, for NTHi-AOM, the expression of PDK1 was compared between the four control samples and 4 NTHi-AOM samples from the GSE27990 dataset, and PDK1 expression was significantly elevated (Figures 4C,D).

### Hub MitoDEGs are associated with immune-infiltration

**FIGURE 5:**
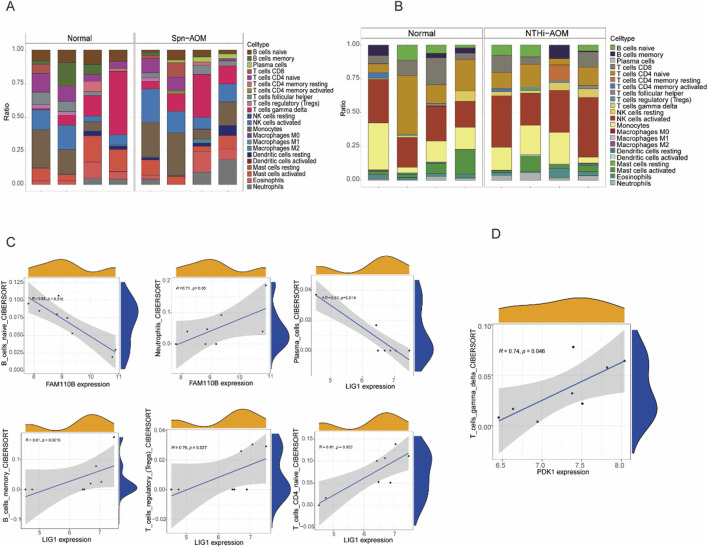
Correlation analysis between hub genes and immune cells. **(A)** Bar chart showing the proportions of immune cell types in Spn-AOM using the Cibersort algorithm. **(B)** Bar chart displaying the proportions of immune cell types in NTHi-AOM using the Cibersort algorithm. **(C)** Correlation analysis between FAM110B and B cells naive, Neutrophils, as well as LIG1 and Plasma Cells, B Cells Memory, T Cells Regulatory, and T Cells CD4 Naive (Spn-AOM). **(D)** Correlation analysis between PDK1 and T Cells Gamma Delta (NTHi-AOM).

Aberrant immune responses contribute significantly to the pathogenesis and progression of acute otitis media (AOM), influencing both infection control and tissue inflammation. Understanding immune cell infiltration in AOM provides valuable insights into host defense mechanisms and potential therapeutic targets. In this study, the immune landscape of AOM and normal samples was assessed via calculating the proportions of 22 immune cell types (Figures 5A,B). In Spn-AOM, FAM110B showed inverse correlation with naïve B cells but demonstrated a positive linkage with neutrophils, whereas LIG1 displayed inverse correlation with plasma cells and positive correlation with memory B cells, regulatory T cells, and naïve CD4^+^ T cells (Figure 5C). Similarly, in NTHi-AOM, PDK1 displays a positive trend with gamma delta T cells (Figure 5D). Together, these findings indicate that mitochondria-related genes FAM110B, LIG1, and PDK1 are key regulators of the immune microenvironment in AOM, potentially influencing disease progression.

### Constructing the TF-miRNA-mRNA network

**FIGURE 6:**
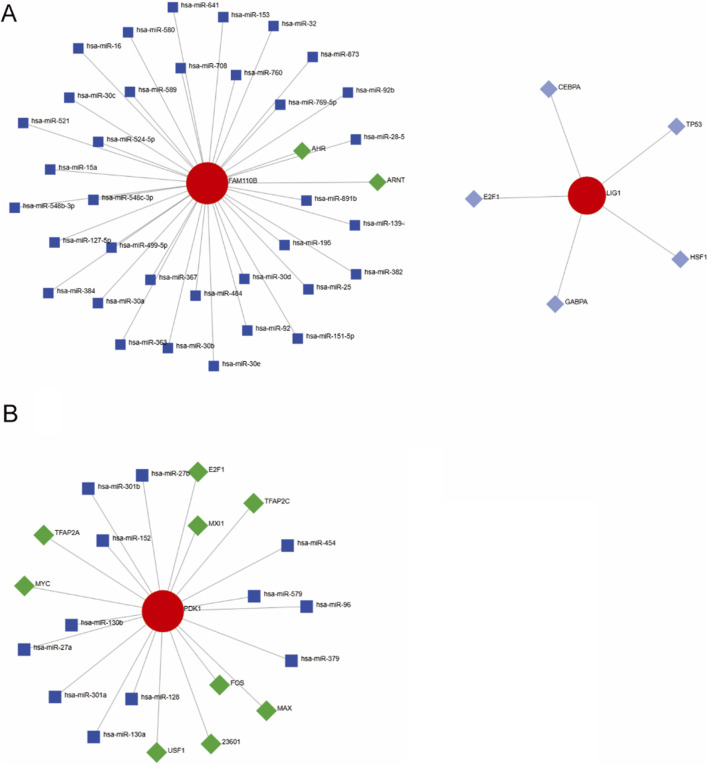
TF-miRNA-mRNA Regulatory Networks. **(A)** Spn-AOM network illustrating interactions between hub genes (red), transcription factors (green), and miRNAs (blue). **(B)** NTHi-AOM network showing interactions among hub genes (red), transcription factors (green), and miRNAs (blue).

Dysregulation of specific transcription factors (TFs) and microRNAs (miRNAs) has been implicated in modulating host immune responses and mitochondrial function during infection. By reconstructing regulatory networks focused on these mitochondrial hub genes, we aimed to identify upstream regulators that may drive their aberrant expression in AOM, thereby providing deeper mechanistic insights into disease-specific gene regulatory circuits. The regulatory mechanisms of mitochondria-related genes in AOM remain unclear, thus generate a TF-miRNA-mRNA network model. As shown in Figure 6A, transcription factors AHR and ARNT, along with miRNAs, were identified as potential regulators of FAM110B in Spn-AOM. Additionally, LIG1 was associated with multiple transcription factors, including E2F1, TP63, HSF1, CEPBA, and GABPA. In NTHi-AOM, PDK1 was found to be regulated by several transcription factors, such as E2F1, MYC, TFAP2A, FOS, MAX, USF1, TFAP2C, and MAX11, along with specific miRNAs (Figure 6B). These findings highlight the potential regulatory roles of transcription factors and miRNAs in the pathogenesis and progression of AOM, offering new perspectives on disease mechanisms and therapeutic targets.

### Verification of mitochondrial-related hub genes in AOM

**FIGURE 7:**
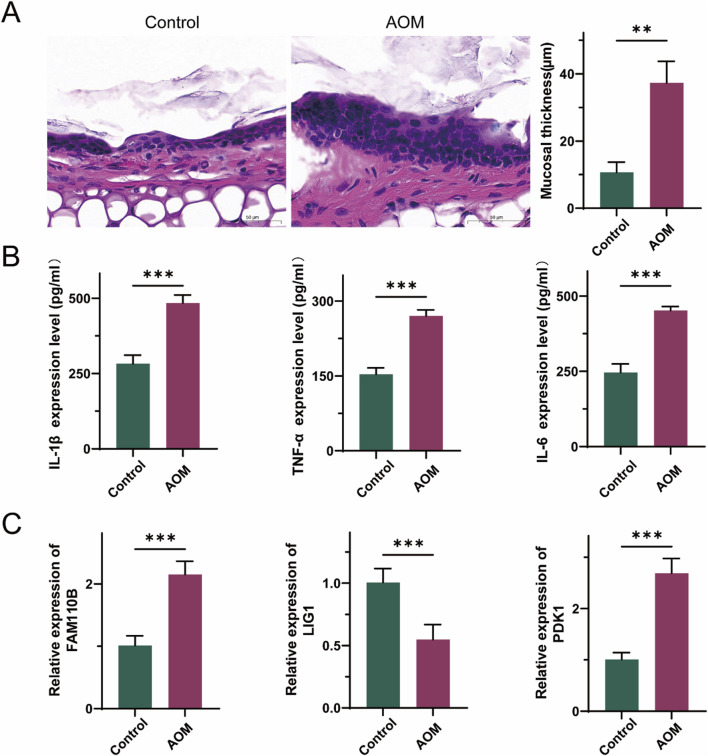
Validation of the expression levels of three hub genes in AOM. **(A)** Representative histological images of middle ear tissues harvested from mice. scale bars = 50 μm. **(B)** The levels of TNF-α, IL-1β and IL-6 in peripheral blood serum were detected by ELISA. **(C)** mRNA levels of three hub genes in peripheral blood serum. (Data are reported as mean ± SEM, n = 6. *P < 0.05, **P < 0.01, ***P < 0.001).

To assess the therapeutic potential of signature genes in AOM, we established an Spn-induced AOM model in C57BL/6 mice. Histological examination of middle ear tissue revealed leukocyte infiltration and increased mucosal thickness in the AOM group (Figure 7A), confirming the successful establishment of the model. H&E staining further highlighted the characteristic pathological features of acute inflammation in the middle ear. Additionally, *Streptococcus* pneumoniae infection was associated with an excessive inflammatory response. Assessment of cytokine concentrations in peripheral blood serum demonstrated a substantial elevation in pro-inflammatory signaling factors such as TNF-α, IL-1β, and IL-6 in AOM group (Figure 7B). Moreover, RT-qPCR was performed to investigate the relationship between AOM and the mRNA expression of hub genes. As illustrated in Figure 7C, FAM110B and PDK1 exhibited significantly higher expression in AOM tissues than in normal tissues, while LIG1 expression was lower, aligning with previous reports.

## Discussion

Acute otitis media (AOM) is one of the most common childhood diseases, often linked to viral, fungal, or bacterial infections. Antibiotics remain the fundamental treatment approach for AOM(5). however, if left untreated, AOM can progress to chronic otitis media, potentially resulting in hearing loss (Borska et al., 2012). Mitochondria plays a crucial role in regulating innate immunity, inflammation, and antibacterial responses (Mills et al., 2017; Nakahira et al., 2011). Despite this, the relationship between mitochondria and AOM remains poorly characterized. To address this gap, we employed bioinformatics analyses to uncover mitochondrial-related gene expression variations in AOM. Our study identified FAM110B, LIG1, and PDK1 as key hub genes in Spn-AOM and NTHi-AOM. Immune infiltration analysis further revealed that these genes are closely associated with immune cell infiltration, underscoring their significance in AOM pathogenesis. Additionally, through TF-miRNA-mRNA network predictions, we proposed a potential regulatory mechanism for these key genes. Collectively, our study offers fresh perspectives on the contribution of mitochondria to AOM.

Mitochondrial dysfunction is a significant factor contributing to the heterogeneity and complexity of individual microenvironments. Our results demonstrate that mitochondrial-related genes such as FAM110B, LIG1, and PDK1 are dysregulated in AOM. Functional enrichment analysis using GO and KEGG pathways further suggests that these genes are involved in mitochondrial matrix regulation, peroxisome function, cell cycle control, and amino acid metabolism and transport. Previous research indicates that dysfunctional mitochondria exacerbate barrier impairment and inflammation, while inflammatory stimuli influence mitochondrial metabolic functions (Li et al., 2018; Ip et al., 2017). Given the established links between AOM, inflammatory responses, and oxidative stress (Li et al., 2018), our findings suggest that mitochondrial-related genes play a pivotal role in AOM progression. It is noteworthy that FAM110B, LIG1, and PDK1 are all protein-coding genes. As hub genes identified through LASSO and RF analyses, they are associated with mitochondrial metabolism and inflammatory pathways. Meanwhile, miRNAs play important roles in AOM by regulating relevant signaling pathways, which provides a perspective at the non-coding RNA level for AOM research.​

Among the identified hub genes, FAM110B and LIG1 were associated with Spn-AOM. FAM110B, a member of the FAM110 family, is a centrosome-associated protein complex component found in both the cytoplasm and mitochondria (Hauge et al., 2007). It has been implicated in cell cycle regulation and tumor progression (Xie et al., 2020). Our study found that FAM110B is significantly upregulated in Spn-AOM, negatively correlated with naïve B cells, and positively correlated with neutrophils, suggesting its involvement in AOM progression. LIG1, a DNA ligase essential for DNA maintenance and repair (Liddiard et al., 2019), is crucial for embryonic development, and mutations in LIG1 have been linked to immunodeficiency (Tumbale et al., 2019). We observed significant downregulation of LIG1 in Spn-AOM, with immune infiltration analysis demonstrating an opposing trend with plasma cells and a positive correlation with memory B cells, regulatory T cells, and naïve CD4^+^ T cells. As no previous studies have explored the roles of FAM110B and LIG1 in AOM, further investigation is required to clarify their functions in Spn-AOM.

PDK1 was identified as the hub gene in NTHi-AOM. PDK1 is a serine/threonine kinase involved in cellular signaling and metabolic regulation. Our study revealed that PDK1 is upregulated in NTHi-AOM and positively correlated with gamma delta T cells. PDK1 is known to inhibit mitochondrial function by phosphorylating and suppressing pyruvate dehydrogenase (PDH), thereby constraining the metabolic shift from pyruvate to acetyl-CoA and restricting mitochondrial energy production (Papandreou et al., 2006; Rankin and Giaccia, 2016). Although a direct link between PDK1 and AOM has not been established, the involvement of mitochondria in immune responses and inflammation suggests that further exploration of PDK1 in AOM pathogenesis is warranted. PDK1 is upregulated in the Spn-AOM mouse model. Although it does not show significant differential expression in the human Spn-AOM dataset, it may serve as a common molecular regulator in AOM of different etiologies and is expected to be a therapeutic target for cross-subtype AOM.​

In conclusion, this study examined the differential expression of mitochondrial-related genes in AOM, identifying key hub genes for Spn-AOM and NTHi-AOM and highlighting their distinct expression patterns in different AOM subtypes. We further validated the expression of these core genes through *in vivo* experiments. However, due to the limitations of a small sample size and the absence of clinical parameter data, we were unable to conduct additional validation of the identified genes. Bacterial and viral AOM share mucosal inflammation and innate immune activation but differ in pathogenesis, with distinct treatments and their own limitations. Future studies incorporating clinical data are necessary to experimentally verify our findings and further elucidate the role of mitochondrial-related genes in AOM.

## Conclusion

In conclusion, this study systematically revealed the critical role of mitochondria-related genes in the pathogenesis of AOM. Using machine learning approaches, we identified key hub MitoDEGs, including FAM110B and LIG1 in Spn-induced AOM and PDK1 in NTHi-induced AOM. These genes appear to regulate immune cell infiltration and modulate mitochondrial function, highlighting their potential as subtype-specific biomarkers and therapeutic targets. Future studies that integrate single-cell transcriptomics, multi-omics analyses, and genetic editing tools will be essential to further elucidate the mechanisms of these MitoDEGs within the immune microenvironment of AOM.

## Data Availability

The datasets presented in this study can be found in online repositories. The names of the repository/repositories and accession number(s) can be found in the article/supplementary material.
